# Mapping malaria risk among children in Côte d’Ivoire using Bayesian geo-statistical models

**DOI:** 10.1186/1475-2875-11-160

**Published:** 2012-05-09

**Authors:** Giovanna Raso, Nadine Schur, Jürg Utzinger, Benjamin G Koudou, Emile S Tchicaya, Fabian Rohner, Eliézer K N’Goran, Kigbafori D Silué, Barbara Matthys, Serge Assi, Marcel Tanner, Penelope Vounatsou

**Affiliations:** 1 Département Environnement et Santé, Centre Suisse de Recherches Scientifiques en Côte d’Ivoire, BP 1303, Abidjan 01, Côte d’Ivoire; 2 Department of Epidemiology and Public Health, Swiss Tropical and Public Health Institute, P.O. Box, CH-4002, Basel, Switzerland; 3 University of Basel, P.O. Box, CH-4003, Basel, Switzerland; 4 UFR Sciences de Nature, Université d’Abobo-Adjamé, 02 BP 801, Abidjan 02, Côte d’Ivoire; 5 Vector Group, Liverpool School of Tropical Medicine, Pembroke Place, Liverpool, L3 5QA, United Kingdom; 6 UFR Biosciences, Université de Cocody, 22 BP 522, Abidjan 22, Côte d’Ivoire; 7 Global Alliance for Improved Nutrition, P.O. Box 55, Rue de Vermont 37-39, CH-1211, Geneva 20, Switzerland; 8 Swiss Centre for International Health, Swiss Tropical and Public Health Institute, P.O. Box, CH-4002, Basel, Switzerland; 9 Programme National de Lutte Contre le Paludisme, BP V4, Abidjan, Côte d’Ivoire; 10 Institut Pierre Richet, 01 BP 1500, Bouaké 01, Côte d’Ivoire

## Abstract

**Background:**

In Côte d’Ivoire, an estimated 767,000 disability-adjusted life years are due to malaria, placing the country at position number 14 with regard to the global burden of malaria. Risk maps are important to guide control interventions, and hence, the aim of this study was to predict the geographical distribution of malaria infection risk in children aged <16 years in Côte d’Ivoire at high spatial resolution.

**Methods:**

Using different data sources, a systematic review was carried out to compile and geo-reference survey data on *Plasmodium* spp. infection prevalence in Côte d’Ivoire, focusing on children aged <16 years. The period from 1988 to 2007 was covered. A suite of Bayesian geo-statistical logistic regression models was fitted to analyse malaria risk. Non-spatial models with and without exchangeable random effect parameters were compared to stationary and non-stationary spatial models. Non-stationarity was modelled assuming that the underlying spatial process is a mixture of separate stationary processes in each ecological zone. The best fitting model based on the deviance information criterion was used to predict *Plasmodium* spp. infection risk for entire Côte d’Ivoire, including uncertainty.

**Results:**

Overall, 235 data points at 170 unique survey locations with malaria prevalence data for individuals aged <16 years were extracted. Most data points (n = 182, 77.4%) were collected between 2000 and 2007. A Bayesian non-stationary regression model showed the best fit with annualized rainfall and maximum land surface temperature identified as significant environmental covariates. This model was used to predict malaria infection risk at non-sampled locations. High-risk areas were mainly found in the north-central and western area, while relatively low-risk areas were located in the north at the country border, in the north-east, in the south-east around Abidjan, and in the central-west between two high prevalence areas.

**Conclusion:**

The malaria risk map at high spatial resolution gives an important overview of the geographical distribution of the disease in Côte d’Ivoire. It is a useful tool for the national malaria control programme and can be utilized for spatial targeting of control interventions and rational resource allocation.

## Background

In 2004, Côte d’Ivoire was ranked at position number 14 with regard to the global burden of malaria with an estimated 767,000 disability-adjusted life years (DALYs) [1]. According to the 2009 World Malaria Report, the estimated population at risk of malaria in Côte d’Ivoire was 21 million people; hence 100% of the population. There were an estimated 1.8 million suspected malaria cases, 33,000 in-patient cases and 18,000 deaths due to malaria [2]. The coverage with insecticide-treated nets (ITNs) has been estimated at only 13% in Côte d’Ivoire, with 6% of the households possessing at least one ITN and 4% of children aged under five years sleeping under an ITN. Although the artemisinin-based combination therapy (ACT) policy has been adopted by the country, these treatments are not yet available free of charge for children aged under five years. Strikingly, due to the post-election crisis starting in late 2010 [3], there was a provisional stopping of support by the Global Fund to Fight AIDS, Tuberculosis and Malaria (Global Fund in short), and hence the planned interventions by the national malaria control programme could not take place. Indeed, the lack of access to appropriate treatment emerged as an urgent public health issue.

With the end of the crisis in mid-2011 and the stability of the country slowly returning [3], the national malaria control programme has re-started its activities, and there is a pressing need for tools that can be implemented rapidly and cost-effectively to mitigate the burden of malaria. High-resolution predictive risk maps can assist authorities to spatially target interventions according to local needs. Such risk maps are typically based on regression models employing climatic and other environmental factors as covariates due to their important role in malaria transmission. A number of studies have shown that *Plasmodium* infections are influenced by environmental factors such as temperature, rainfall, humidity and elevation [4-8]. These factors directly or indirectly influence the development and occurrence of *Anopheles* mosquitoes, the malaria vectors, and hence, affect the geographical distribution of malaria.

Standard statistical modelling approaches assume independence between survey locations and neglect potential spatial dependency between neighbouring locations due to unobserved common exposures. Geo-statistical models take into account spatial correlation by additional location-specific random effect parameters. These models have already been applied to model malaria risk at different geographical scales in sub-Saharan Africa [5,9-19].

In Côte d’Ivoire, there is considerable climatic variation from north to south, leading to the sub-division of the country into different ecological zones. Isotropic geo-statistical models assume that spatial correlation is a function of distance between locations irrespective of locations themselves. However, spatial correlation might vary across the country due to the presence of different ecological regions, variation in health system performance, socio-economic differentials or intervention coverage, introducing non-stationarity. Non-stationary models had previously been applied to model malaria risk in western Côte d’Ivoire, Mali and western sub-Saharan Africa [12-14].

Given the need and the lack of contemporary national malaria surveys in Côte d’Ivoire (i.e. malaria indicator surveys – MIS), this work used a geo-statistical modelling approach for point-prevalence data from a wide array of sources for children under the age of 16 years to predict malaria risk in Côte d’Ivoire, including uncertainty measures. The modelling approach was adjusted for key environmental risk factors.

## Methods

### Study area

Côte d’Ivoire is located in West Africa and has an area of 322,462 km^2^. It borders the countries of Liberia and Guinea in the west, Mali and Burkina Faso in the north and Ghana in the east. The total population is estimated at 21 million. The climate of Côte d’Ivoire is generally warm and humid, ranging from equatorial in the southern coasts and tropical in the centre to semi-arid in the far north. There are three seasons: warm and dry (November to March), hot and dry (March to May), and hot and wet (June to October). The temperature averages between 25°C and 32°C and ranges from 10°C to 40°C. The south-eastern part of Côte d’Ivoire is marked by coastal inland lagoons that start at the Ghanaian border and stretch 300 km along the eastern half of the coast. The southern region, especially the south-west, is covered with dense tropical moist forest. The Guinean forest-savannah mosaic belt extends across the middle of the country from east to west. The northern part of Côte d’Ivoire belongs to the West Sudanian savannah.

### Data sources

A systematic search was carried out on PubMed to identify all surveys that reported *Plasmodium* spp. prevalence data for Côte d’Ivoire. The authors’ own bibliographies were also systematically searched. Additionally, a broad-based search of grey literature was conducted, including local journals, MSc and PhD theses from national universities and libraries of research institutes, Ministry of Health (MoH) reports and personal communication. The period covered was between 1988 and 2007. In case geographical coordinates of malaria point prevalence data were missing from the literature, the locations were searched on a map and the respective coordinates extracted. Whenever possible, authors were contacted for provision of supplementary information on the reported data. Given that most of the studies focused on children, it was decided that for modelling purposes, only prevalence data for children aged <16 years were to be assembled. In case older age groups were also sampled in a particular study, the authors were asked to provide individual level data, so that the prevalence for the target age group (i.e. <16 years) could be extracted. For each data point, the age-specific number of children examined and the percentage tested positive for *Plasmodium* spp. infection were extracted. Additionally, the year when the survey was carried out was recorded. The prevalence data used in this study are available upon request from the authors.

Elevation (altitude above sea level, expressed in m) was obtained from USGS EROS data centre digital elevation model (DEM) at a spatial resolution of 1 km. Distance to the nearest water body (in m) was computed based on the Health Mapper data files for rivers, lakes and wetlands. Summarized estimates for eight-day maximum land surface temperature (LST) and 16-day normalized difference vegetation index (NDVI) were obtained from Moderate Resolution Imaging Spectroradiometer (MODIS) at a spatial resolution of 1 km during the period from 2000 to 2008. Ten-day rainfall data were obtained from the Africa Data Dissemination Service (ADDS) at a spatial resolution of 8 km. At each location the mean annual maximum temperature, mean annual rainfall, elevation and distance to water bodies were extracted.

Ecological zones were derived by importing rainfall, elevation, NDVI, land cover and maximum temperature data into ERDAS Imagine 9.3 software. An unsupervised classification via the iterative self-organizing data analysis technique (ISODATA) was used on the above ecological factors to create three different ecological zones based on between-class similarities. Centroids of each ecological zone in Côte d’Ivoire were derived via ArcMap version 9.2 (ESRI) for subsequent modelling purposes.

### Statistical analysis

Binomial regression models were fitted in STATA/IC version 10.1 (StataCorp LP; College Station, TX, USA) to assess the relation between ecological predictors and *Plasmodium* spp. prevalence. Significant ecological factors, based on likelihood ratio test (LRT) with significance levels of 15%, were included as covariates in further analyses. Bayesian non-spatial and geo-statistical logistic regression models were fitted in OpenBUGS version 3.0.3 (Imperial College and Medical Research Council; London, UK). Spatial dependency was modelled assuming stationary (i.e. spatial correlation was modelled as a function of distance between locations only), as well as non-stationary (i.e. spatial correlation was modelled as a function of distance between locations and position within the study area) latent spatial processes.

### Model formulation

Let *N*_*i*_ be the number of children tested at location *s*_*i*_ (i =1, …, *n*) and *Y*_*i*_ the number of those found with *Plasmodium* parasites in a blood sample. It was assumed that *Y*_*i*_ arises from a binomial distribution, that is *Y*_*i*_ ~ *Bin*(*N*_*i*_, *p*_*i*_), with *p*_*i*_ measuring malaria risk at location *s*_*i*_. The relation between the malaria risk and the *m* associated environmental covariates *X*_*i*_ at location *s*_*i*_*, X*_*i*_ = (*X*_*i1*_, *X*_*i2*_, …, *X*_*im*_)^*T*^, was modelled via the logistic regression *logit*(*p*_*i*_) = *X*_*i*_^*T*^*ß*, where *ß* = (*ß*_*1*_, *ß*_2_, …, *ß*_*p*_)^*T*^ are the regression coefficients. Exchangeable random effects *ϵ*_*i*_ were added on the logit scale, such as *logit*(*p*_*i*_) = *X*_*i*_^*T*^*ß + ϵ*_*i*_.

Spatial correlation was introduced on location-specific random effect parameters *φ*_*i*_, that is log*it*(*p*_*i*_) = *X*_*i*_^*T*^*ß* + *φ*_*i*_, assuming that *φ* = (*φ*_*1*_, *φ*_*2*_, …, *φ*_*n*_)^*T*^ ~ *MVN*(0,Σ) with variance-covariance matrix Σ. It was further assumed that spatial process is isotropic and decays exponentially with distance, i.e. Σ_*ij*_ = *σ*^*2*^exp(−*ρd*_*ij*_), where *d*_*ij*_ is the Euclidean distance between villages *s*_*i*_ and *s*_*j*_; *σ*^*2*^ is the geographic variability known as sill, and *ρ* is a smoothing parameter that controls the rate of correlation decay with increasing distance. The spatial range is defined as the minimum distance at which spatial correlation between locations is below 5%, and is calculated as 3/*ρ* for the exponential correlation structure.

To take into account non-stationarity, the study area was partitioned into three ecological sub-regions (*K* = 3), assuming local independent stationary spatial processes *ω*_*k*_ = (*ω*_*k1*_*ω*_*k2*_, …, *ω*_*kN*_)^*T*^ in each ecological sub-region (*k* = 1, …, *K*). The spatial processes were assumed to be multi-variate normally distributed, *ω*_*k*_ ~ *MVN*(0,Σ_*k*_), with variance-covariance matrixes Σ_*k*_ defined by (Σ_*k*_)_*ij*_ = *σ*^*2*^_*k*_*exp*(*ρ*_*k*_*d*_*ij*_). It was further considered that the spatial correlation *φ*_*i*_ at location s_*i*_ in the study area is a mixture of the independent spatial processes modelled as weighed average, such as $\varphi_{i}=\sum_{k=1}^{K}a_{\mathrm{ik}}\omega_{\mathrm{ik}}$, where the weights *a*_*ik*_ are decreasing functions of the distance between location *s*_*i*_ and the centroids of the sub-regions *k*[20,21]. Under these specifications, *φ* follows a multivariate normal distribution, *φ* ~ *MVN*(0,$\sum_{k=1}^{K}A_{k}^{T}\Sigma_{k}A_{k}$), where *A*_*k*_ = *diag*(*a*_*1k*_*a*_*2k*_, …, *a*_*nk*_).

In a Bayesian modelling framework, specification of prior distributions of all model parameters is required. Vague normal priors with large variance were assumed for the *β* parameters, while inverse gamma priors were chosen for *σ*^2^ and *σ*_*k*_^2^ and uniform priors for *ρ* and *ρ*_*k*_. Markov chain Monte Carlo (MCMC) simulation was employed to estimate the model parameters [22]. A single chain sampler with a burn-in of 2,000 iterations was run for around 100,000 iterations. Convergence was assessed by inspection of ergodic averages of selected model parameters. The deviance information criterion (DIC) was used to assess the goodness-of-fit of the models without and with exchangeable random effects, and the stationary and non-stationary geo-statistical models [23]. The smaller the DIC, the better the model fit. Finally, Bayesian kriging was used to generate smooth risk maps for *Plasmodium* infection prevalence based on the parameter estimates of the best fitting model [24].

## Results

### Identified studies and description of georeferenced survey data

**Figure 1:**
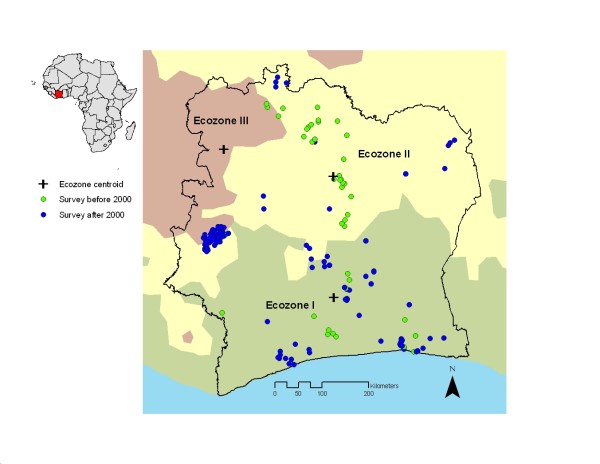
** Geographical distribution of *Plasmodium *spp. surveys locations based on compiled data on children aged <16 years between 1988 and 2007 in Côte d’Ivoire.** The data were stratified in two categories: survey carried out before and since the year 2000. The extent of three major ecozones in the country, which was derived from various satellite data, is displayed in the background. Centroids of the ecozones are given as black symbols.

The systematic and broad-based search revealed a total of 29 data sources (17 peer-reviewed articles, six theses, four reports and two personal communications) for a 20-year period starting in 1988. A total of 235 data points with malaria prevalence data for individuals aged <16 years (at 170 unique survey locations) were extracted, of which 53 (22.6%) pertained to surveys carried out between 1988 and 1999, and the remaining 182 data points (77.4%) were collected between 2000 and 2007. A third of the data points (n = 80, 34.0%) were located in the region of Man in western Côte d’Ivoire arising from two large cross-sectional surveys carried out in 2001 and 2003/2004. Figure 1 shows the spatial distribution of data points within Côte d’Ivoire and the extent of the derived ecozones in the background, stratified by survey year. Ecozone III had considerably fewer data points, most of which were concentrated at the southern border of the zone. In contrast, in ecozones I and II, data points were well distributed across the regions.

### *Plasmodium* spp. prevalence

**Figure 2:**
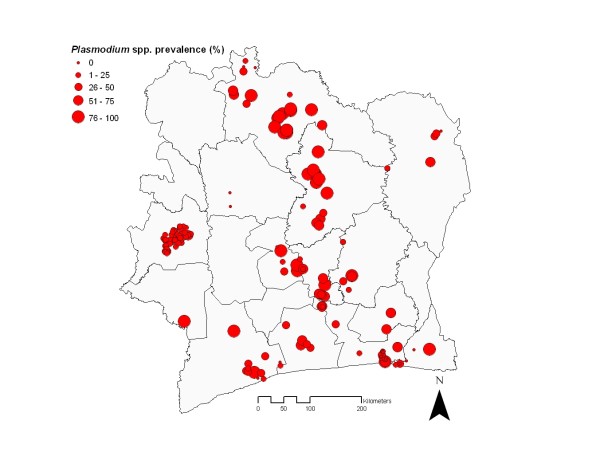
** Map of *Plasmodium *spp. prevalence obtained from surveys on children aged <16 years carried out between the years 1988 and 2007 in Côte d’Ivoire.**

For subsequent logistic regression analyses, data on *Plasmodium* spp. prevalence were used. Surveys with missing information on *Plasmodium* spp. was assumed as *Plasmodium falciparum* prevalence since other species, i.e. *Plasmodium ovale* and *Plasmodium malariae*, are much less frequent in Côte d’Ivoire [25-30]. The *Plasmodium* prevalence in children <16 years ranged from nil to 100% with a mean prevalence of 54.1%. The observed prevalence at the data points distributed over the country is shown in Figure 2. In south Côte d’Ivoire, *Plasmodium* prevalence is generally lower compared to the rest of the country. Indeed, when subdividing Côte d’Ivoire into three equally large strata from lowest to highest latitude, namely south, central and north, the mean prevalence of *Plasmodium* was 37.3%, 61.5% and 61.0%, respectively.

### Non-spatial regression analysis

Table 1 shows the results of the non-spatial and spatial logistic regression analyses. The non-spatial multivariate model (model 1) revealed that elevation and distance to closest rivers were significantly positively associated with *Plasmodium* spp. prevalence, while rainfall and maximum LST were negatively associated. When introducing exchangeable random effects (model 2), model performance based on DIC estimates improved considerably (9,974 *vs.* 1,501). The random effect had also an influence on the regression parameters of the covariates. Association with distance to rivers became non-significant, while stronger negative effects of the covariates rainfall and maximum LST on the outcome were observed.

**Table 1:** **Parameter estimates based on logistic regression models for *Plasmodium *spp. prevalence in children aged <16 years in Côte d’Ivoire using compiled data from surveys carried out between 1988 and 2007**

	Non-spatial model		Non-spatial model with exchangeable random effects		Stationary spatial model		Non-stationary spatial model	
Model parameter	(*Model 1*)		(*Model 2*)		(*Model 3*)		(*Model 4*)	
	OR^a^	BCI^b^	OR^a^	BCI^b^	OR^a^	BCI^b^	OR^a^	BCI^b^
Elevation	1.44	1.40, 1.48	1.07	1.01, 1.13	1.05	0.99, 1.11	1.05	0.98, 1.11
Distance to rivers	1.08	1.06, 1.11	1.02	0.94, 1.10	0.98	0.91, 1.06	0.98	0.91, 1.05
Mean rainfall	0.91	0.88, 0.94	0.73	0.67, 0.79	0.77	0.70, 0.83	0.76	0.70, 0.83
Mean maximum LST^c^	0.89	0.86, 0.92	0.67	0.60, 0.74	0.72	0.65, 0.80	0.72	0.64, 0.79
*σ*^*2*^			1.59	1.20, 2.09	2.37	1.29, 4.74		
*σ*^*2*^_*1*_*(ecozone I)*							1.56	0.78, 2.67
*σ*^*2*^_*2*_*(ecozone II)*							4.76	2.05, 10.49
*σ*^*2*^_*3*_*(ecozone III)*							0.10	0.006, 0.40
*ρ*					1.98	0.70, 3.82		
*ρ*_*1*_*(ecozone I)*							39.44	12.02, 59.35
*ρ*_*2*_*(ecozone II)*							1.55	0.52, 3.23
*ρ*_*3*_*(ecozone III)*							27.61	2.30, 57.62
***DIC***^d^	**9,974**		**1,501**		**1,485**		**1,479**	

^a^*OR* Odds ratio; ^b^*BCI* Bayesian credible interval; ^c^*LST* Land surface temperature; ^d^*DIC* Deviance information criterion.

### Spatial regression analysis

The introduction of location-specific stationary random effect parameters into the model (model 3) showed a strong leverage on model performance and parameter estimates compared to model 2. Elevation became non-significantly related to the outcome, leaving the model with only rainfall and maximum LST as significant covariates. The model fit based on DIC estimates improved considerably (1,485). When looking at the results of the non-stationary model (model 4), the DIC decreased further (1,479), suggesting that this is the best model. Of note, for model 4 the covariates elevation and distance to closest rivers were non-significant as well, whereas rainfall and maximum LST remained significant, as it had been observed for the other models.

Model 3 estimated a larger geographic variability *σ*^*2*^ compared to the non-spatial model 2 (2.37 *vs*. 1.59). The geographic variability *σ*_2_^2^ estimated for ecozone II from the non-stationary model was particularly large compared to *σ*_1_^2^ and *σ*_3_^2^. The estimated spatial range (above which spatial correlation drops below 5%) from the stationary model 3 was about 151 km. However, taking into account non-stationarity, the spatial range varied between 8 km (ecozone I) and 193 km (ecozone II).

### Risk mapping

**Figure 3:**
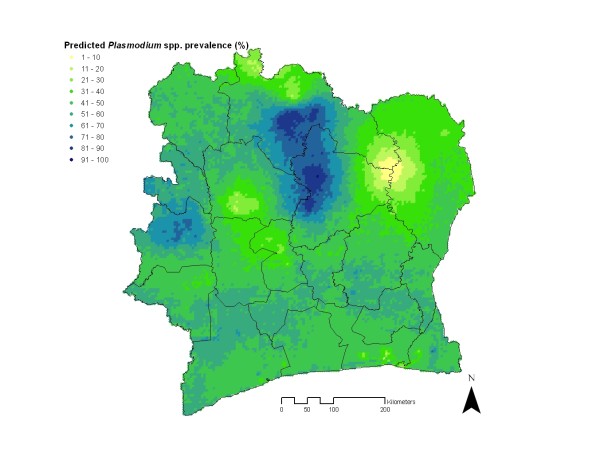
** Smoothed risk map of *Plasmodium *spp. infection for children aged <16 years in Côte d’Ivoire using a Bayesian non-stationary logistic regression model.**

Figure 3 shows the predicted malaria risk map for Côte d’Ivoire as obtained from the best fitting model, namely the non-stationary logistic regression model (model 4). Highest malaria prevalence (>70%) was predicted in the north-central part and in the western part of Côte d’Ivoire. In contrast, low prevalence estimates (≤30%) were predicted for the following areas: (i) in the north at the country border, (ii) in the north-east, (iii) in the south-east around the economic capital Abidjan, and (iv) in the central-west between two high prevalence areas. Noteworthy, model prediction of the largest low prevalence area occurred in the north-east, but the model estimates in this area were based on a single data point.

### Uncertainty of the malaria risk map for Côte d’Ivoire

**Figure 4:**
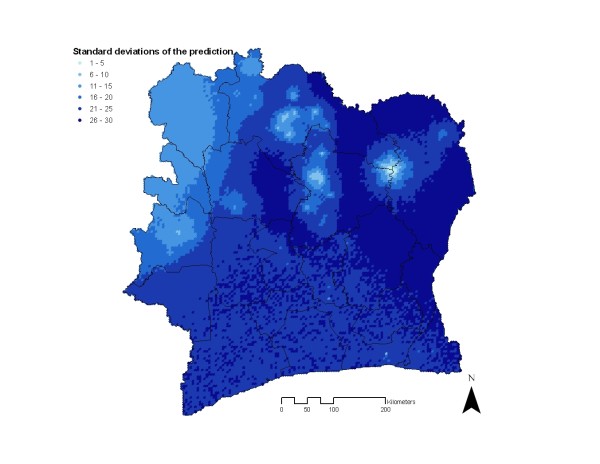
** Map of the standard deviation of model-based predictions of *Plasmodium *spp. infection risk inferred from the Bayesian non-stationary logistic regression model.**

The standard deviation map of the predictive posterior distribution obtained from model 4 is given in Figure 4. As expected, it depicts low prediction error around the survey locations. For instance, in the north-eastern part of Côte d’Ivoire where low prevalence of *Plasmodium* spp. was predicted, the error rapidly increases with increasing distance from survey locations. This area in the north-east of Côte d’Ivoire has only few survey data, and hence standard deviations were overall high.

## Discussion

Most malaria-related deaths, morbid sequelae and economic losses could be averted through implementation of control measures with a proven track record, such as ITNs, indoor residual spraying (IRS) and access to prompt diagnosis and treatment using ACT to those populations in highest need [31]. Since 2007 and until the post-election crisis that emerged in November 2010, malaria control in Côte d’Ivoire has been largely funded by the Global Fund. However, much remains to be done to significantly reduce the burden of malaria through large-scale implementation of control interventions [2]. It is conceivable that the socio-political conflict and unrest that prevailed for most of the past 12 years, hindered progress on malaria control among other pressing public health issues [3,32,33]. Indeed, during the most recent armed conflict, malaria interventions were interrupted altogether. Now that political stability has resumed, there is a pressing need to re-establish and strengthen health systems, preventive measures and disease control programmes. The aim of this study was to provide a country-wide map of the geographical distribution of malaria risk that could help in the spatial planning of future control interventions by the national malaria control programme, readily adapted to local needs and current capacities.

Previous efforts obtained global, regional or continent-wide estimates, which included Côte d’Ivoire [5,10]. However, these previous estimates captured the variation of malaria risk at large spatial scales, and the accuracy at high resolution may be compromised. In fact, countries with the same climatic conditions may not have the same disease burden because country-specific factors (e.g. different intervention coverage, performance of health systems) may modify the risk. Indeed, in several areas of Côte d’Ivoire, the health system was heavily affected or partially interrupted in the last decade [32]. The malaria risk map presented in this article shows that there is spatial variation in the prevalence of *Plasmodium* spp. infection. This is also observed in the newly produced global *P. falciparum* map, but differences are apparent, especially with regard to zones where the present model predicts low prevalence [34]. Another previous map produced at regional scale for West and Central Africa also revealed spatial variation [5], but differences, especially for low prevalence rates (<30%), can also be observed with the current map.

Overall, 235 geo-referenced malaria prevalence survey data points were obtained. Bayesian geo-statistical methods were used to model the point prevalence data, using logistic regression models with key environmental covariates derived from remotely sensed data. Two non-spatial and two spatial models were fitted with elevation, distance to rivers, rainfall and maximum LST employed as environmental covariates. For the spatial models, the assumption of stationarity was extended to non-stationarity of the spatial process, in line with previous work [11-14,21,35]. Ecological zoning was considered to partition the study area into meaningful sub-regions with locally distinct ecological characteristics. Indeed, the models employed here confirmed that spatial correlation differed depending on the location (ecozone) and that the non-stationary spatial model was superior to the remaining models in predicting *Plasmodium* spp. prevalence.

In the spatial models, the covariates rainfall and maximum LST were significantly associated with *Plasmodium* prevalence. These environmental factors had already been successfully implemented in previous geo-statistical modelling approaches employed in other African countries [5,8,13,36]. Côte d’Ivoire has spatially distinct climate conditions that vary from equatorial in the south coast, to tropical in the centre, and semi-arid in the north, with more rainfall in the south compared to the north, and higher temperature amplitudes in the north compared to the south, which might explain the significant contribution that these covariates had in the spatial models. Although other environmental covariates such as elevation and distance to rivers were significant in the non-spatial models, they became non-significant in the spatial models. This demonstrates the importance of accounting for spatial correlation, when analysing geographical data in order to avoid over-estimation of the standard errors of model covariates [37]. Topographically, Côte d’Ivoire can be considered as a vast plateau, with exception of the west of the country (Dix-Huit Montagnes region), which has mountains with peaks of 1,000 m and above (the highest elevation is Mount Nimba, 1,752 m above sea level, located along the border with Guinea). Previous work from this region demonstrated that elevation, rainfall and temperature have no influence on the spatial distribution of *Plasmodium* prevalence at small scale [14]. The current study confirms that the effect of elevation on malaria prevalence at country-level is insignificant for Côte d’Ivoire. However, rainfall and temperature were identified to be important drivers of *Plasmodium* spp. prevalence at a larger scale. Of note, rainfall was found to drive *Plasmodium* parasitaemia also at small scale in the mountainous part of western Côte d’Ivoire [13].

The present results are based on compiled survey data assembled for Côte d’Ivoire covering a 20-year period until 2008. There are several issues related to the use of assembled data worth discussing. First, the distribution of survey data was very rich, especially in the west (region of Man), south and central part of the country, while data availability was extremely sparse in the north-west and north-east. Hence, the predicted prevalence estimates should be interpreted with caution and in combination with the map of uncertainty (Figure 4). For instance, the model predicted low *Plasmodium* prevalence in the north-east of Côte d’Ivoire based on only one data point and, given the high spatial correlation and the scarcity of data in this region (also due to low population density), the model predicted a relatively large area with low prevalence, which might not reflect the real situation. Fortunately, Bayesian geo-statistical modelling allows inferring measures of uncertainties from the model-based predictions, which can help in the interpretation of the risk maps. Second, many of the surveys lack *Plasmodium* species-specific information and therefore it was decided to model *Plasmodium* spp. rather than species-specific prevalence. Malaria, however, is not a single disease and there are five *Plasmodium* species that cause human malaria, which are transmitted by over 30 *Anopheles* mosquito species [38]. This entails different disease spectra in different population target groups from different epidemiological settings, with implications for current malaria control programmes [38]. Hence, future studies need to make an effort to consistently and correctly report species-specific information on *Plasmodium* prevalence in order to improve control. Despite this shortcoming, it must be mentioned that in Côte d’Ivoire, *P. falciparum* is the predominant species, as consistently shown across the country [25-30]. Hence, the *Plasmodium* spp. risk map presented here is closely imitating a *P. falciparum* risk map. Third, the models were not adjusted for age although it is well acknowledged that malaria prevalence differs between age groups [39]. Therefore, future modelling should take into account the age-prevalence-relation via mathematical transmission models [40,41] converting age-heterogeneous survey data to a common measure which is used for mapping purposes [9,10]. Fourth, remotely sensed climate data (rainfall and maximum LST) were used to calculate mean values over the period from 2000 to 2008, although obtained prevalence data dated back to 1988. This period was chosen because MODIS data (maximum LST) were not available before the year 2000, and alignment with historical temperature data was not successful. Although it might be expected that models with year-specific climate variables could result in better model fits, the use of mean climatic values reduces the effect of abnormal climatic conditions that might have occurred during the study period, avoiding artefacts in the parameter estimates.

## Conclusion

Although the need for evaluating the value of detailed disease incidence and prevalence maps to inform programmatic responses in evaluation and surveillance at a global scale has been expressed, malaria risk mapping at national level is crucial to support and plan interventions according to local needs in countries where control or elimination strategies are underway [42]. Côte d’Ivoire is currently focusing efforts on scaling up malaria control interventions. The current malaria risk map and future maps taking into account the latest prevalence data can provide detailed information on transmission changes and assist in monitoring and evaluation of current control activities of the re-established malaria control programme.

In the past, at present and in the future, *Plasmodium* spp. risk maps have guided and will continue to guide decision makers in Côte d’Ivoire and elsewhere for spatial targeting of malaria control activities, e.g. assisting estimates on case management and related procurement of ACT and rapid diagnostic tests and where to prioritize ITN and IRS activities. Although risk maps using historical data have to be interpreted with caution, Bayesian geo-statistical risk mapping provides information on the uncertainty of the model-based malaria risk estimates. Nonetheless, the results indicate that there is a need of more detailed malaria prevalence data in Côte d’Ivoire, preferentially obtained from a national survey on randomly selected locations, with species-specific information. Furthermore, the usability of routine data collected by a re-invigorated health system in Côte d’Ivoire should be explored without delay. Future risk mapping approaches might be improved by including information on intervention coverage, vector distribution and human population density, distribution and movement patterns.

## Abbreviations

ACT: Artemisinin-based combination therapy; ADDS: Africa Data Dissemination Service; BCI: Bayesian credible interval; DALY: Disability-adjusted life year: DEM, Digital elevation model; DIC: Deviance information criterion; ITN: Insecticide-treated net; ISODATA: Iterative self-organizing data analysis technique; LST: Land surface temperature; MCMC: Markov chain Monte Carlo; MIC: Malaria indicator survey; MODIS: Moderate Resolution Imaging Spectroradiometer; NDVI: Normalized difference vegetation index.

## Competing interests

The authors declare that they have no competing interests.

## Authors’ contributions

GR, NS, BGK and EST performed the systematic searches and geo-referenced malaria survey data. GR, JU, BGK, FR, EKN, KDS, SA and BM contributed malaria survey data. GR and NS carried out the spatial analyses and interpretation of the data and drafted the manuscript. JU, BGK, EST, KDS, FR, EKN, SA, BM, MT and PV assisted with the interpretation of the data and the revision of the manuscript. All authors read and approved the final manuscript.
